# Identification of the Specific Interactors of the Human Lariat RNA Debranching Enzyme 1 Protein

**DOI:** 10.3390/ijms16023705

**Published:** 2015-02-09

**Authors:** So Masaki, Rei Yoshimoto, Daisuke Kaida, Asuka Hata, Takayuki Satoh, Mutsuhito Ohno, Naoyuki Kataoka

**Affiliations:** 1 Medical Innovation Center, Laboratory for Malignancy Control Research, Kyoto University Graduate School of Medicine, Sakyo-ku, Kyoto 606-8507, Japan; E-Mails: masaki@dsk.med.kyoto-u.ac.jp (S.M.); akusa.atah@gmail.com (A.H.); 2 Chemical Genetics Laboratory, RIKEN Advanced Science Institute, Wako, Saitama 351-0198, Japan; E-Mail: ryoshi@fujita-hu.ac.jp; 3 Institute for Virus Research, Kyoto University, Sakyo-ku, Kyoto 606-8507, Japan; E-Mail: hitoohno@virus.kyoto-u.ac.jp; 4 Division of Gene Expression Mechanism, Institute for Comprehensive Medical Science, Fujita Health University, 1-98 Dengakugakubo, Kutsukake-cho, Toyoake, Aichi 470-1192, Japan; 5 Frontier Research Core for Life Sciences, University of Toyama, 2630 Sugitani, Toyama-shi, Toyama 930-0194, Japan; E-Mails: kaida@med.u-toyama.ac.jp (D.K.); tsatoh@med.u-toyama.ac.jp (T.S.); 6 Medical Top Track Program, Medical Research Institute, Tokyo Dental and Medical University, Tokyo 113-8510, Japan

**Keywords:** pre-mRNA splicing, intron turnover, RNA lariat, debranching, hDbr1, Xab2, hDrn1

## Abstract

In eukaryotes, pre-mRNA splicing is an essential step for gene expression. We have been analyzing post-splicing intron turnover steps in higher eukaryotes. Here, we report protein interaction between human Debranching enzyme 1 (hDbr1) and several factors found in the Intron Large (IL) complex, which is an intermediate complex of the intron degradation pathway. The hDbr1 protein specifically interacts with xeroderma pigmentosum, complementeation group A (XPA)-binding protein 2 (Xab2). We also attempted to identify specific interactors of hDbr1. Co-immunoprecipitation experiments followed by mass spectrometry analysis identified a novel protein as one of the specific interactors of hDbr1. This protein is well conserved among many species and shows the highest similarity to yeast Drn1, so it is designated as human Dbr1 associated ribonuclease 1 (hDrn1). hDrn1 directly interacts with hDbr1 through protein–protein interaction. Furthermore, hDrn1 shuttles between the nucleus and the cytoplasm, as hDbr1 protein does. These findings suggest that hDrn1 has roles in both the nucleus and the cytoplasm, which are highly likely to involve hDbr1.

## 1. Introduction

Most protein-coding genes encoded in the nucleus are separated by introns in higher eukaryotes [1,2]. Pre-mRNA splicing, a process to remove introns and ligate exons, is an essential process for gene expression. Introns are removed from pre-mRNA by a large RNA–protein complex, the spliceosome, as a lariat form [3,4,5,6]. Excised introns are thought to be retained and degraded in the nucleus. This step has not been well analyzed compared with the splicing reaction in the spliceosome. However, it is likely that this step is important, especially in higher eukaryotes, because many small nucleolar RNAs (snoRNAs) and microRNAs (miRNAs) are encoded in introns and the processing steps of these noncoding RNAs are coupled with splicing reactions [7,8,9,10,11,12]. Before the degradation of lariat introns, a 2'–5' phosphodiester bond formed between branch point adenosine residue and the guanosine residue of the 5' splice site is dissolved. This reaction is catalyzed by an RNA lariat debranching enzyme, Dbr1 [13,14,15,16]. The Dbr1 protein is well conserved among many organisms and a GNHE motif, which is also found in some protein phosphatases, is a catalytic center of this protein [14,16]. The human Dbr1 protein, hDbr1, was shown to shuttle between the nucleus and the cytoplasm, while the steady state localization of this protein is in the nucleoplasm, suggesting a new function of hDbr1 in the cytoplasm [16].

We have been analyzing the post-splicing intron turnover steps with a human splicing reaction system. We found that the intron degradation pathway includes two steps with two RNA-protein complexes [17]. The first one is the Intron Large (IL) complex, which consists of a lariat intron RNA, U2, U5 and U6 snRNPs, and several splicing factors. The other complex is called the Intron Small (IS) complex, which has no detectable UsnRNPs and less protein than the IL complex. The hDbr1 protein can debranch a lariat RNA in the IS complex, but not in the IL complex [17]. This strongly suggests that the branch point region of lariat RNA is covered in the IL complex and becomes accessible after transition to the IS complex. The transition from the IL to the IS is mediated by the human Prp43 (hPrp43) protein and its binding partner TFIP11/hNtr1 [17]. As an interactor to both hPrp43 and TFIP11, we identified C2ORF3 protein, which is a new component of the IL complex [18]. Since hDbr1 protein was not detected in the IL and the IS complexes [17], the association of hDbr1 with lariat intron is likely to be transient. Although hDbr1 could debranch lariat introns in the IS complex, it was not able to debranch them in the IL complex [17]. These results strongly suggest that a factor(s) in the IL complex inhibits hDbr1 association with the intron lariat complex. Recently, it has been reported that Drn1 binds to Dbr1 and modulates its debranching activity in yeast [19], but the specific interactors of human Dbr1 were not identified. In the present study, we analyzed the protein-protein interaction between human Dbr1 and the factors found in the IL complex, and found that xeroderma pigmentosum, complementeation group A (XPA)-binding protein 2 (Xab2) can interact with hDbr1 among them. We also attempted to identify the specific interactors of hDbr1 and identified a novel protein called Cwf19L1. This protein shows high similarity to yeast Drn1, and binds to hDbr1 *in vitro* and *in vivo*. Furthermore, hDrn1 shuttles between the nucleus and the cytoplasm, suggesting a role in the cytoplasm that is likely to involve hDbr1.

## 2. Results

### 2.1. The hDbr1 Protein Interacts with Xab2

**Figure 1:**
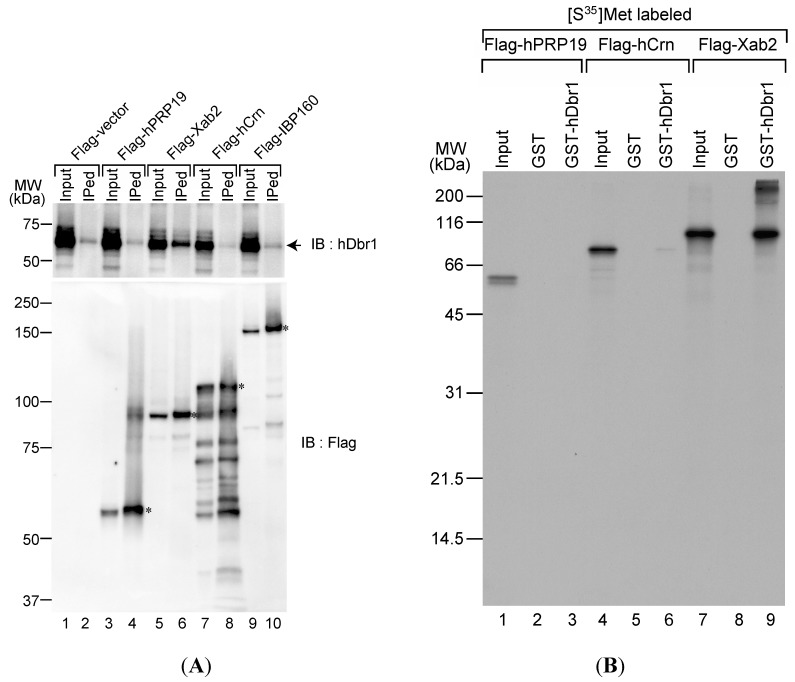
The hDbr1 protein associates with xeroderma pigmentosum, complementeation group A (XPA)-binding protein 2 (Xab2) *in vivo* and *in vitro*. (**A**) The *in vivo* association of several IL complex proteins with hDbr1 protein was analyzed by immunoprecipitation assay. HEK293T whole-cell extract was prepared after transfection with Flag-vector, Flag-hPrp19, Flag-Xab2, Flag-hCrn or Flag-IBP160 plasmid. Flag-tagged proteins were precipitated using anti-Flag M2 agarose from whole-cell extract, and the precipitates (IPed) were subjected to Western blotting analyses using anti-hDbr1 antibody (**upper** panel) and an anti-Flag polyclonal antibody (**lower** panel). A portion (5%) of the precipitated proteins (lanes marked as Input) was also immunoblotted. The position of endogenous hDbr1 protein is indicated by an arrow in the upper panel. The full-length Flag-tagged proteins are marked with asterisks in the lower panel. The positions of the protein size markers are described on the left of the panel; and (**B**) *In vitro* binding of hDbr1 to Xab2. Flag-tagged hPrp19, hCrn and Xab2 proteins, produced and labeled with [^35^S]methionine *in vitro*, were incubated with 5 μg each of GST or GST-hDbr1. After precipitation and washing, bound proteins were eluted, separated on 12.5% SDS-PAGE gel and visualized by fluorography. The lanes marked Input contain 10% of the total protein used in each binding reaction. The position of molecular mass markers is shown on the left of the panel.

We previously identified the components of the IL complex, which is an intermediate complex for post-splicing intron turnover steps [17]. Although hDbr1 could debranch lariat introns in the IS complex, it was not able to debranch them in the IL complex [17]. These results strongly suggest that factor(s) in the IL complex inhibits hDbr1 association with the intron lariat complex and the IS complex factor(s) confers hDbr1 association on it. However, the factors in the IS complex have yet to be identified [17]. Therefore, we attempted to determine interactions between the IL complex proteins and hDbr1, because the IL complex likely contains the IS complex components. We carried out immunoprecipitation experiments with Flag-tagged IL complex proteins followed by Western blotting using specific antibodies against hDbr1 protein. We transfected cDNAs encoding hPrp19, Xab2, hCrn and IBP160 into HEK293T cells. Flag-vector was used as a control. As shown in Figure 1A, transfected cDNAs were successfully expressed in HEK293T cells and Flag-tagged proteins were produced (lower panel). We carried out immunoprecipitation from total cell lysates by using anti-Flag M2 antibody to test the immunoprecipitation efficiency. The antibody precipitated full-length Flag-tagged proteins from the lysates (Figure 1A, lower panel). Under these conditions, anti-hDbr1 antibody was used to test whether or not endogenous hDbr1 protein is co-precipitated with those proteins. Figure 1A demonstrates that only Flag-Xab2 precipitated endogenous hDbr1 protein (upper panel, lane 6). We then tested protein–protein interaction between Xab2 and hDbr1 *in vitro*. *In vitro* translated proteins were incubated with either GST or GST-hDbr1 protein and bound proteins were analyzed by SDS-PAGE. As shown in Figure 1B, *in vitro* translated Xab2 bound to GST-hDbr1, while hPRP19 did not bind and hCrn bound very weakly (lanes 3, 6 and 9). Taken together, these results indicate that hDbr1 can interact with Xab2 among the IL complex components we tested.

### 2.2. Identification of a Novel Protein as a Specific Interactor to hDbr1

**Figure 2:**
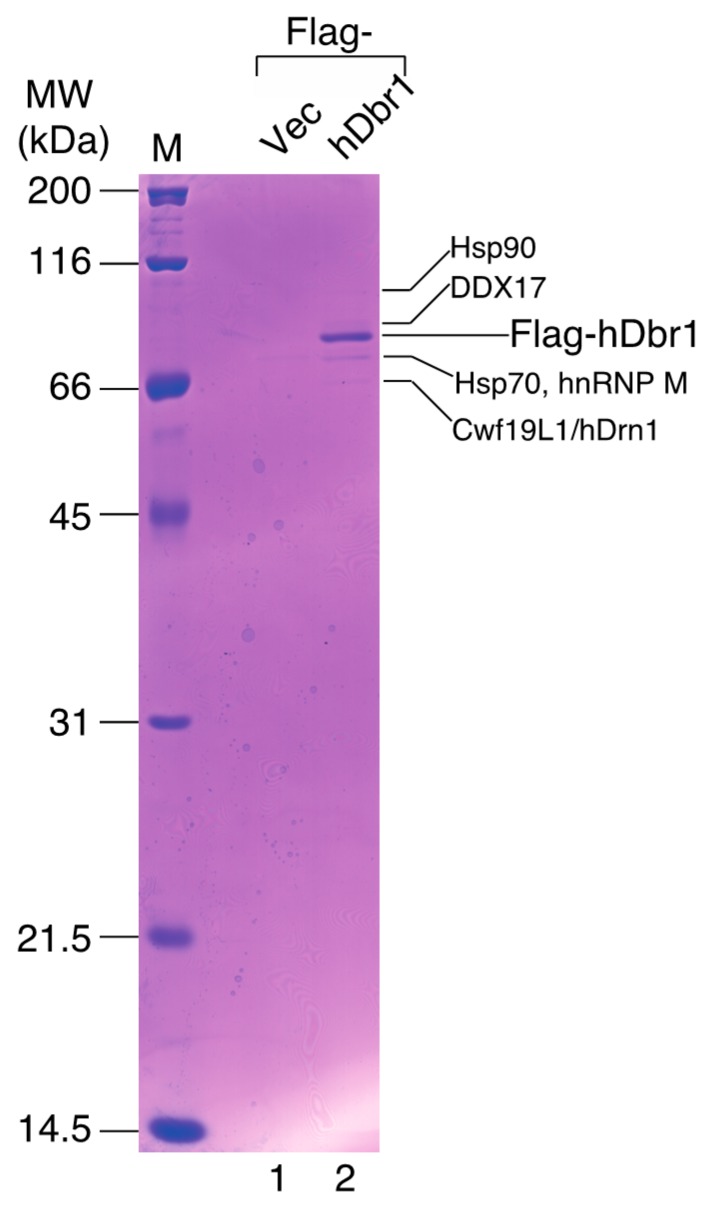
Identification of the proteins interacting with hDbr1 by MALDI-TOF mass spectrometry. Immunoprecipitation of the hDbr1 complexes with an antibody against Flag tag (M2; Sigma, St. Louis, MO, USA) from HEK293T cell extracts overexpressing Flag-hDbr1 (lane 2). The whole-cell extract prepared from the Flag-pcDNA3-transfected cells was used as a control (Flag-Vec, lane 1). Proteins were separated by 12.5% SDS-PAGE and visualized by Coomassie Brilliant Blue staining. The proteins identified by MALDI-TOF mass spectrometry are indicated by their names. The positions of the size marker proteins are indicated by their sizes (kDa) on the left.

**Figure 3:**
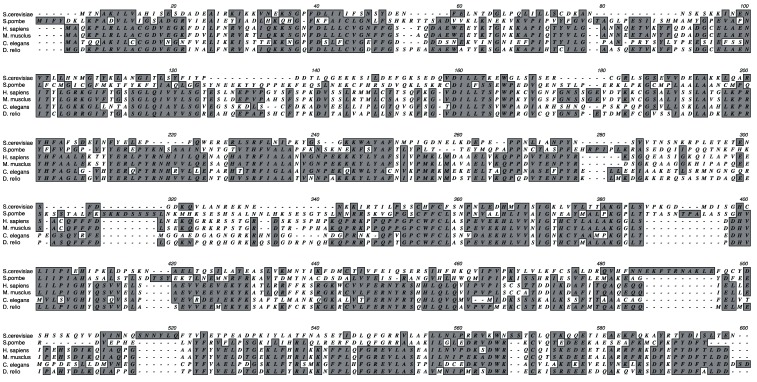
Amino acid sequence alignment of Drn1 proteins from various organisms. Drn1 proteins from *Saccharomyces cerevisiae* (NP_011607.3), *Schizosaccharomyces pombe* (NP_593012.1), *Homo sapiens* (NP_060764.3), *Mus musculus* (NP_001074546.1), *Caenorhabditis elegans* (NP_504577.1) and *Danio rerio* (NP_001038223.1) were aligned using the ClustalW alignment program (http://www.genome.jp/tools/clustalw/). Identical and similar residues are indicated by dark shading.

In order to identify more specific binders to hDbr1, we carried out co-immunoprecipitation experiments followed by mass spectrometry analysis. Flag-tagged hDbr1 protein was expressed in HEK293T cells and precipitated by anti-Flag M2 resin. After spectrometry analysis, several proteins were identified, as shown in Figure 2 (lane 2). Among the interactors, we found an unknown protein, which has recently been designated as Cwf19L1 [20]. We searched for possible orthologs of Cwf19L1 in other organisms using protein databases and found that this protein is well conserved among many species, from budding yeast to humans, suggesting its important role(s) (Figure 3), since it shows high homology to the yeast *Ygr093w* gene product. This gene is also described as debranching enzyme-associated ribonuclease 1 in the *Saccharomyces* gene database (SGD) [19], so we designated it as human Drn1 (hDrn1) and further characterized the interaction between hDbr1 and hDrn1. HSP70 and HSP90 were also identified as the interactors to hDbr1 in Figure 2 (lane 2). Although these two proteins were identified in lariat-intron complex [17], we found that these proteins also bind to other overexpressed proteins and MS2 protein likely non-specifically (data not shown). Thus, we did not analyze these proteins further in this work.

### 2.3. The hDrn1 Protein Binds Specifically and Directly to hDbr1

**Figure 4:**
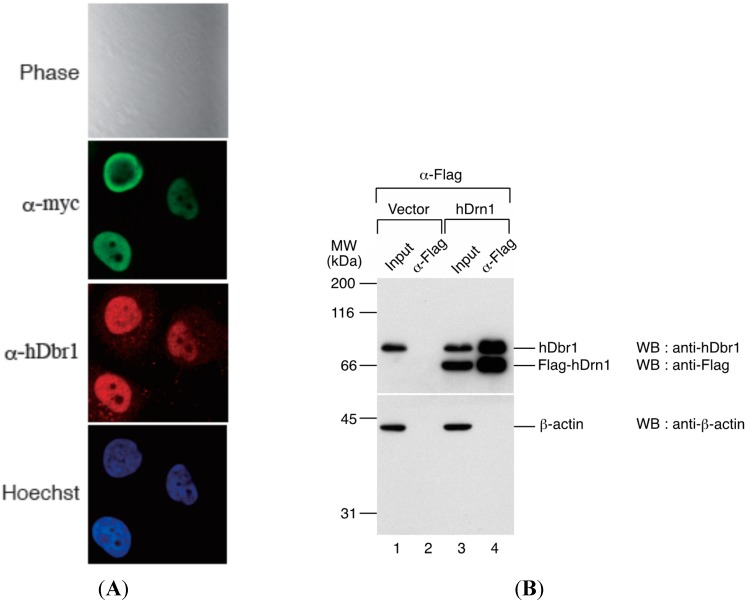
hDrn1 localizes to the nucleoplasm in HeLa cells by immunofluorescence and co-immunoprecipitates with endogenous hDbr1. (**A**) The expression vector encoding myc-tagged hDrn1 was transfected into HeLa cells. Twenty-four hours after transfection, the cells were stained with anti-myc (M192-3, MBL, panel FITC, **middle**), anti-hDbr1 16019-1-AP, Proteintech) and Hoechst 33342 (SIGMA, panel Hoechst, **bottom**) to label the nuclei. Differential interference contrast (DIC) image of the cells is also shown as Phase panel at the **top**; and (**B**) Co-immunoprecipitation of endogenous hDbr1 with Flag-hDrn1 *in vivo*. Flag-tagged hDrn1 was expressed in HEK293T cells and immunoprecipitated using anti-Flag M2 antibody (SIGMA). Co-precipitated fractions were separated using 12.5% SDS-PAGE gel and analyzed by Western blotting using anti-hDbr1 and anti-Flag polyclonal antibodies (**upper** panel) and anti-β-actin antibody (MBL) (**lower** panel). Input lanes contain 5% of the total proteins used for immunoprecipitation assays. The positions of protein mass markers are shown on the left in kDa.

**Figure 5:**
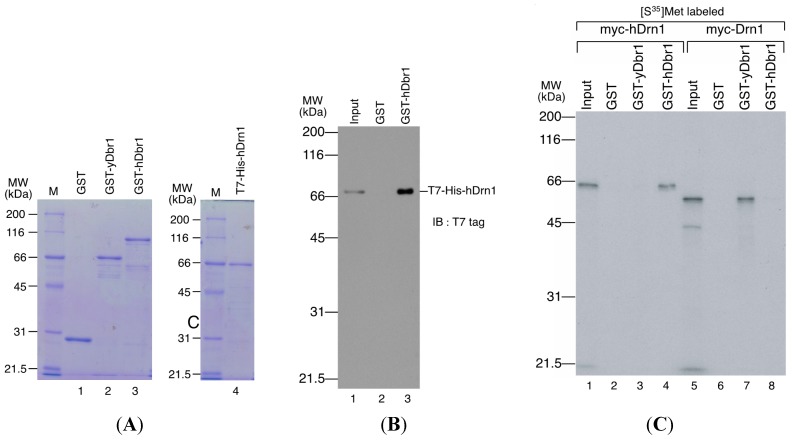
hDrn1 binds directly with hDbr1 and the binding of these proteins is conserved among species. (**A**) Proteins used for *in vitro* binding assays. GST, GST-yeast Dbr1 (GST-yDbr1), GST-hDbr1 and His-hDrn1 were overexpressed and purified from *E. coli* cells. Two micrograms of each protein was separated using 12.5% SDS-PAGE gels and stained with Coomassie Brilliant Blue. The molecular mass markers are also shown on the left of the gels with the sizes in kDa; (**B**) Direct binding of hDrn1 with hDbr1 *in vitro*. Two micrograms of purified His-tagged hDrn1 was incubated with 5 μg each of GST or GST-hDbr1. Bound proteins were separated by 12.5% SDS-PAGE and detected by Western blotting with anti-T7 tag antibody (Novagen). The molecular mass markers are also shown on the left of the gels; and (**C**) Specific binding between Dbr1 and Drn1 is conserved between yeast and humans. The myc-tagged yDrn1 and hDrn1 proteins were produced and labeled with [^35^S]methionine *in vitro*. Those proteins were incubated with 5 μg each of GST, GST-yDbr1 or GST-hDbr1. After precipitation and washing, bound proteins were eluted, separated on 12.5% SDS-PAGE gel and visualized by fluorography. The lanes marked Input contain 10% of the total protein used in each binding reaction. The position of the molecular mass markers is shown on the left of the panel in kDa.

We first determined the subcellular localization of hDrn1 in HeLa cells. The hDrn1 protein was expressed as a fusion protein with myc tag peptide in HeLa cells and immunofluorescence analysis was carried out by using anti-myc tag antibody. As shown in Figure 4A, myc-hDrn1 was localized mainly in the nucleoplasm of HeLa cells and some nucleolus signals could be detected (α-myc panel), which is similar to that of hDbr1 (α-hDbr1 panel). The anti-hDbr1 antibody also exhibited some dot-like signals in the cytoplasm, whose nature is currently unknown (α-hDbr1 panel). Next, we tested whether hDrn1 interacts with hDbr1 *in vivo*. Flag-tagged hDrn1 protein was expressed in HEK293T cells by transfection and hDrn1 was immunoprecipitated using anti-Flag M2 resin. Flag-pcDNA3 vector was used as a negative control. The precipitated fractions were analyzed by Western blotting. The results in Figure 4B show that endogenous hDbr1 protein was specifically and efficiently precipitated by Flag-hDrn1, while β-actin was not (Figure 4B, lane 4). We also confirmed the specific binding of hDrn1 to hDbr1 *in vitro*. The recombinant Dbr1 protein was expressed in *E. coli* cells and purified as a fusion protein to GST (Figure 5A, lane 3). GST-fused yeast Dbr1 protein and GST protein were also prepared for binding assays (Figure 5A, lanes 1 and 2). For the direct binding assay, we prepared T7- and His-tagged hDrn1 protein (Figure 5A, lane 4). In order to test whether hDrn1 interacts with hDbr1 directly or not, we carried out *in vitro* binding assays using recombinant proteins. T7-His-tagged hDrn1 protein was mixed with GST-hDbr1 protein, and GST was used as a negative control. The results in Figure 5B indicate that hDrn1 interacts with hDbr1 directly through protein-protein interaction (lane 3). Since hDrn1 showed high similarity to yeast Drn1, we then checked whether the binding between Drn1 and Dbr1 was conserved in these two organisms or not. GST-yDbr1, GST-hDbr1 and GST proteins were incubated with either yDrn1 or hDrn1 prepared by *in vitro* translation. As shown in Figure 5C, human Drn1 bound to GST-hDbr1, but not to yeast Dbr1 and GST (lanes 2–4). In contrast, yeast Drn1 interacted with GST-yDbr1, and did not bind to GST-hDbr1 and GST (Figure 5C, lanes 6–8). These results indicate that the specific interaction between Dbr1 and Drn1 is conserved in yeast and humans, but human protein does not interact with yeast protein and *vice versa*.

**Figure 6:**
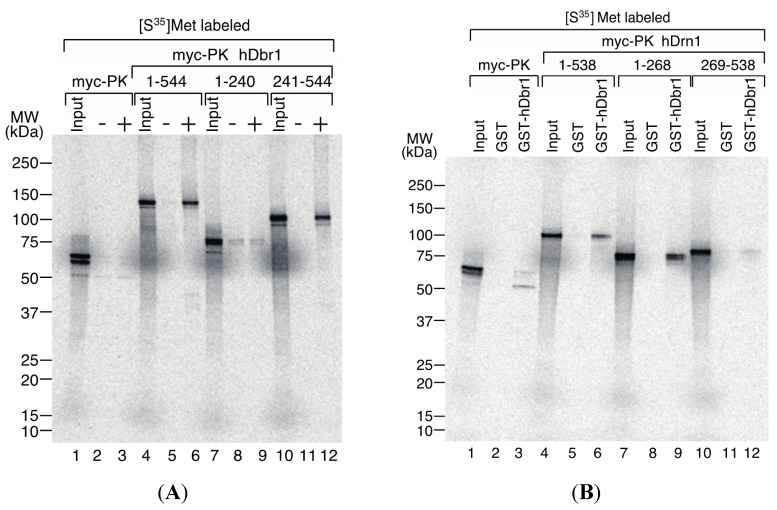
Determination of the interaction domains of hDbr1 and hDrn1. (**A**) 2 μg of T7-His-hDrn1 proteins were incubated with *in vitro* translated myc-PK, myc-PK hDbr1 (aa. 1–544), myc-PK hDbr1 (aa. 1–240) and myc-PK hDbr1 (aa. 241–544). After washing, bound proteins were eluted, separated on 5%–20% gradient SDS-PAGE and visualized by fluorography. Input lanes contain 5% of the total protein used in each binding experiment. The positions of protein molecular mass markers are shown on the left of the panel; and (**B**) *In vitro* translated myc-PK, myc-PK hDrn1 (aa. 1–538), myc-PK hDrn1 (aa. 1–268) and myc-PK hDrn1 (aa. 269–538) were incubated with 5 μg of either GST alone or GST-hDbr1. The bound proteins were analyzed and visualized as in (**A**). Input lanes contain 5% of the total protein used in each binding experiment. The positions of protein molecular mass markers are shown on the left of the panel.

We then tried to determine the interaction domains of hDbr1 and hDrn1 by *in vitro* binding experiments. For the delineation of the binding domain of hDbr1 to hDrn1, T7-His-hDrn1 protein, which was used for the experiments in Figure 5, was employed. The T7-His-hDrn1 proteins immobilized by anti-T7 tag antibody on protein A beads were incubated with *in vitro* translated proteins that have several different portions of hDbr1 protein fused with myc-Pyruvate Kinase (PK) [16]. The myc-PK protein was used as a negative control (lanes 1–3). As shown in Figure 6A, myc-PK hDbr1 full length (aa. 1–544) was able to bind to T7-His-hDrn1, while myc-PK alone showed slight binding as the non-specific level (lanes 4–6 and 1–3, respectively). Carboxy-terminal region (aa. 241–544) of hDbr1 bound to T7-His-hDrn1, but amino-terminal portion (aa. 1–240) of hDbr1, which has a catalytic domain for debranching, exhibited weak binding nonspecifically (lanes 10–12 and 7–9, respectively). These results indicate that the binding of hDbr1 to hDrn1 is mostly conferred by carboxy-terminal region. We next investigated which domain of hDrn1 has the binding activity to hDbr1 *in vitro*. Either full-length (aa. 1–538), amino-terminus (aa. 1–268) or carboxy-terminus (aa. 269–538) of hDrn1 was fused to myc-PK. Those proteins were produced by *in vitro* translation and incubated with either GST alone or GST-hDbr1 protein shown in Figure 5A. Figure 6B demonstrated that both the full-length and amino-terminus of hDrn1 bound to GST-hDbr1, but not to GST alone (lanes 4–6 and 7–9). In contrast, carboxy-terminus of hDrn1 exhibited very weak binding to GST-hDbr1 as myc-PK alone did (lanes 10–12 and 1–3, respectively). Taken together, amino-terminus region of hDrn1 mostly mediates the binding to hDbr1, which is consistent with the previously reported results with Drn1 by yeast two-hybrid method [19].

### 2.4. The hDrn1 Protein Has Shuttling Activity between the Nucleus and the Cytoplasm

**Figure 7:**
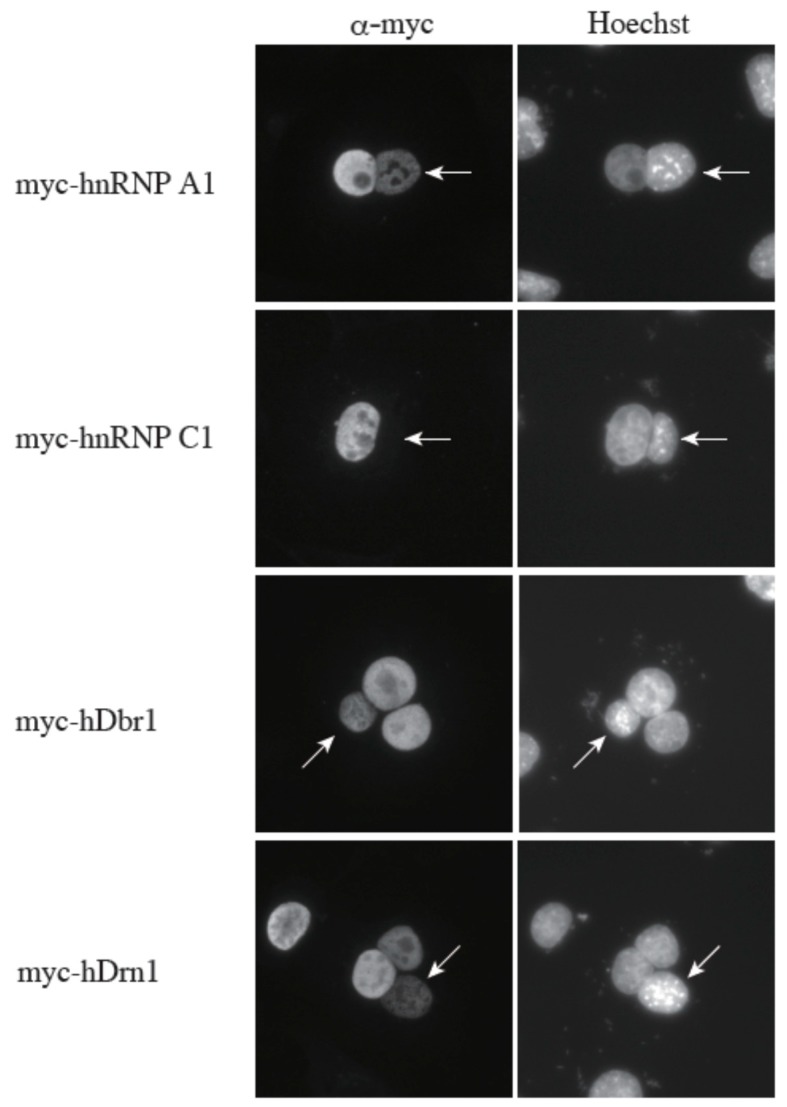
The hDrn1 protein shuttles between the nucleus and the cytoplasm as hDbr1. Expression vectors encoding hnRNP A1, C1, hDbr1 and hDrn1 with myc-tag were transfected into HeLa cells. After expression of the transfected cDNAs, the cells were fused using PEG with mouse NIH3T3 cells to form heterokaryons and incubated in culture medium containing 100 μg/mL cycloheximide for two hours. The heterokaryon cells were then fixed and stained for immunofluorescence microscopy with anti-myc tag antibody (panel FITC) to localize the proteins, and Hoechst 33342 (panel Hoechst), which distinguishes the human and mouse nuclei within the heterokaryon. The arrows indicate the mouse nuclei.

We previously demonstrated that hDbr1 shuttles between the nucleus and the cytoplasm [16]. Since hDrn1 binds to hDbr1, we hypothesized that hDrn1 also has nucleo-cytoplasmic shuttling activity. In order to test this hypothesis, we performed a heterokaryon assay (Figure 7). Heterokaryons were formed by polyethylene glycol (PEG)-mediated fusions of HeLa cells that had been transfected with the indicated constructs in advance and mouse NIH3T3 cells. Staining of the cells with a DNA dye, Hoechst 33342, allowed us to distinguish between the HeLa and the NIH3T3 nuclei. After cell fusion, subsequent incubations were carried out in the presence of cycloheximide to ensure that the observed signals result from the proteins in the HeLa nuclei and not from the newly synthesized proteins in the cytoplasm. A construct containing the rapid shuttling protein hnRNP A1 was a positive control, while the non-shuttling hnRNP C1 protein served as a negative control. The results shown in Figure 7 demonstrate that hDrn1 shuttles between the nucleus and the cytoplasm, since the myc-hDrn1 signal was clearly detected in both HeLa and NIH3T3 nuclei, as was that of hDbr1. These results strongly suggest that hDrn1 has cytoplasmic function(s), likely involving hDbr1.

## 3. Discussion

In this study, we analyzed specific binders of the hDbr1 protein. Previously, we reported the Intron Large (IL) complex as an intermediate of the intron degradation pathway [17]. Among the tested factors in the IL complex, Xab2 could interact with hDbr1 both *in vivo* and *in vitro* (Figure 1). We could not identify Xab2 protein by mass spectrometry analysis of Flag-hDbr1 interacting proteins (Figure 2). As shown in Figure 1A, the co-precipitated amount of hDbr1 protein with Xab2 is low (less than 5%). It is possible that co-precipitated Xab2 with Flag-hDbr1 was below detection level with Coomassie Brilliant Blue staining. The biological function hDbr1 to Xab2 is currently unknown. Since these two factors are essential for viability [21,22,23], it is not possible to completely deplete them from cells. Either knockdown of them by stably expressing shRNA or search for chemical compounds that blocks this interaction has to be carried out to determine the function of this interaction in several biological processes such as splicing and transcription-coupled DNA repair.

We also identified a novel interactor of hDbr1. This protein is called Cwf19L1 in a database, but its molecular function was unknown. It can directly interact with and co-immunoprecipitate with hDbr1 (Figure 2, Figure 4B and Figure 5). Cwf19L1/hDrn1 exhibits a similar nucleoplasm localization pattern to hDbr1 (Figure 4A). These results strongly suggest the role(s) of Cwf19L1/hDrn1 in post-splicing intron turnover with hDbr1. Recently, Drn1, a homolog of RNA lariat debranching enzyme 1 in *Saccharomyces cerevisiae*, was reported to associate with the lariat intron containing splicing products and modulate lariat intron turnover with Dbr1 [19]. Since hDrn1 is likely to be an ortholog of this protein, we attempted to determine whether it can associate with splicing products produced by *in vitro* splicing and whether it enhances/inhibits the debranching activity of hDbr1 *in vitro*. However, we could not detect hDrn1 association with splicing products *in vitro* (data not shown). Consistent with this, we could not find hDrn1 in IL complex [17]. We could not detect any effects of hDrn1 on RNA lariat debranching activity by hDbr1 *in vitro*, neither (data not shown). One possible explanation for these discrepancies are the differences of splicing mode and quality control process by the spliceosome between budding yeast and humans. Another possibility is that the hDrn1 protein has different functions in cells, although it has high similarity to the yeast Drn1 protein. The hDrn1 protein may have function(s) in the cytoplasm, since it can shuttle between the nucleus and the cytoplasm, as hDbr1 does (Figure 7). Several lines of evidence have suggested that lariat introns of certain genes exist and are likely to be degraded in the cytoplasm [24,25,26,27]. In addition, a subset of circular RNAs (circRNAs) was recently reported as a large class of regulatory RNAs, and some of them are localized in the cytoplasm [28,29,30,31,32]. Since it turned out that circRNA biogenesis requires canonical splicing signals and flanking intronic sequences [28,29,30,31,32], intron turnover pathway factors including hDbr1 may be involved in circRNA degradation. The hDrn1 may shuttle between the nucleus and the cytoplasm as a heterodimer and modulate the debranching activity of hDbr1 in the cytoplasm. In order to clarify the possibilities described above, further experiments including knockout of hDrn1 in mammalian culture cells and *in vitro* splicing reaction using nuclear extracts from those cells are required.

## 4. Experimental Section

### 4.1. Plasmid Construction

Cwf19L1/hDrn1 cDNA (NM_018294) was amplified by PCR and inserted between *BamH*I and *Xho*I sites of myc- and Flag-pcDNA3 plasmids [33,34] to prepare myc-hDrn1 and Flag-hDrn1 plasmids, respectively. In order to prepare His-tagged hDrn1 protein expression, the same fragment was inserted between *BamH*I and *Xho*I sites of the pET-28a plasmid (Novagen, Madison, WI, USA). Yeast Ygr093w/Drn1 cDNA (NM_001181222) was amplified by PCR and cloned between *BamH*I and *Xba*I sites of myc-pcDNA3 to prepare the myc-DrnI plasmid. GST-hDbr1 was prepared by inserting hDbr1 cDNA (NM_016216) into the *BamH*I and *Xho*I sites of the GEX-6P-1 plasmid (GE Healthcare, Buckingham, UK). Flag-hDbr1, myc-PK and myc-PK fused to several portions of hDbr1 were as described elsewhere [10,16]. Full-length, amino-terminus and carboxy-terminus portions of hDrn1 were amplified by PCR and inserted between *Kpn*I and *Xho*I sites of myc-PK plasmid [16]. In order to prepare Flag-hPrp19, Flag-IBP160, Flag-Xab2 and Flag-hCrn, these cDNA fragments (hPrp19: NM_014502, IBP160: NM_014691, Xab2: NM_020196, hCrn: NM_016652) were amplified by PCR with *BamH*I and *Xho*I enzyme digestion sites. After digestion with those restriction enzymes, the fragments were inserted between *BamH*I and *Xho*I sites of Flag-pcDNA3.

### 4.2. Protein Overexpression and Purification 

Proteins were overexpressed in the BL21 (DE3) CodonPlus-RIPL *Escherichia coli* strain (Agilent Technologies, Palo Alto, CA, USA) and were purified by methods recommended by the manufacturers. Glutathione-*S*-transferase (GST)-fusion proteins were purified with Glutathione Sepharose (GE Healthcare) as per the manufacturer’s protocol. His-tagged proteins were purified using Ni-NTA resin (Novagen) as per the manufacturer’s protocol.

### 4.3. In Vitro Protein Binding

*In vitro* protein binding experiments were performed as described previously [35,36]. Briefly, 5 μg of GST fusion protein was immobilized on glutathione beads (GE Healthcare). For T7-His-hDrn1 binding, 2 μg of anti-T7 tag antibody (Novagen) was immobilized on 5 μL magnetic protein A beads (Tamagawa Seiki, Iida, Japan) in PBS. After wash with 500 μL of PBS, 2 μg of T7-His-hDrn1 was added and immobilized. *In vitro* translated proteins were prepared using a TNT-coupled rabbit reticulocyte lysate system (Promega, Madison, WI) in the presence of [^35^S]methionine. Approximately 2 × 10^5^–3 × 10^5^ c.p.m. (count per minute) of *in vitro* translated product was added to the beads in 500 μL of the binding buffer (50 mM Tris-HCl pH 7.4, 200 mM NaCl, 2 mM EDTA, 0.1% NP-40) and mixed at 4 °C for 1 h. The beads were then washed five times with binding buffer, and bound proteins were eluted with sample buffer and analyzed by SDS-PAGE.

### 4.4. Cell Culture and Transfection

HeLa cell culture and HEK293T cell (American Type Culture Collection (ATCC), Manassas, VA, USA) culture were performed as described previously [33,37]. As a transfection reagent, Lipofectamine2000 (Invitrogen, Carlsbad, CA, USA) was used according to the manufacturer’s protocol.

### 4.5. Immunoprecipitation of Proteins

HEK293T cells grown in two 10-cm dishes were harvested in 1 μL of ice-cold buffer E (20 mM Tris-HCl (pH 8.0), 100 mM KCl, 0.2 mM EDTA, 0.2 mM PMSF) 48 h after the transfection. The cells were sonicated on ice and centrifuged in microcentrifuge tubes for 20 min at 15,000× *g* at 4 °C. The supernatant was incubated with 50 μL of anti-Flag antibody conjugated to agarose beads (anti-Flag M2 affinity gel; Sigma, St. Louis, MO, USA) with constant rotation for 60 min at 4 °C. The beads were washed five times in buffer E and dissolved in the sample buffer for SDS-PAGE. Proteins were analyzed using 12.5% SDS-PAGE followed by Coomassie Brilliant Blue staining.

### 4.6. Mass Spectrometric Analysis

Mass spectrometric identification of proteins was carried out as previously described [17]. After separation by SDS**-**PAGE, the proteins were visualized by Coomassie Brilliant Blue staining. The excised protein bands were digested and subjected to matrix-assisted laser desorption/ionization time-of-flight mass spectrometry (MALDI-TOF/MS) by Invitrogen. Proteins were identified by comparing the molecular weights determined by MALDITOF/MS and the theoretical peptide masses of proteins registered in the NCBInr database using MASCOT software (Matrix Science, London, UK; http://www.matrixscience.com/search_form_select.html).

### 4.7. Western Blotting

Western blotting was carried out as described previously [38]. The antibodies used were as follows: anti-Flag M2 monoclonal antibody (Sigma; 1:500 dilution), anti-hDbr1 [17] (1:1000), anti-T7 tag antibody (1:1000 dilution; Novagen), anti-β-actin (1:1000 dilution; Medical & Biological Laboratories (MBL), Nagoya, Japan), peroxidase-conjugated Affinipure goat anti-mouse IgG + IgM (H + L) (Jackson ImmunoResearch, West Grove, PA, USA) and peroxidase-conjugated Affinipure goat anti-rabbit IgG (H + L) (1:10,000 dilution; Jackson ImmunoResearch).

### 4.8. Indirect Immunofluorescence Analysis

Transfected HeLa cells were fixed and stained for immunofluorescence microscopy as described previously [16,33]. The used antibodies were as follows: anti-myc antibody (Nacalai Tesque) and FITC-conjugated anti-rabbit antibody (Jackson Immunochemicals). Hoechst 33342 was obtained from Life Technologies (Gaithersburg, MD, USA). The images were obtained using a LSM780 System (Carl Zeiss, Oberkochen, Germany) equipped with a 32-Channel GaAsP spectral detector (QUASAR, detection unit, Carl Zeiss).

### 4.9. Heterokaryon Assay

The heterokaryon assays were performed as described previously [16,33,39,40,41]. The co-cultures of HeLa cells and mouse NIH3T3 cells were incubated for 3 h in the presence of 75 μg/mL cycloheximide and then fused with polyethylene glycol (PEG) as described previously [16,33,39,40,41]. The heterokaryons were returned to DMEM medium containing 100 μg/mL cycloheximide and incubated for 2 h before fixation for indirect immunofluorescence analysis.
